# The effect of the clinical supervision model on nursing internship students’ nursing process-based performance: an experimental study

**DOI:** 10.1186/s12912-024-01840-0

**Published:** 2024-03-08

**Authors:** Amir Shahzeydi, Sedigheh Farzi, Mohammad Javad Tarrahi, Fakhri Sabouhi, Sima Babaei, Ahmadreza Yazdannik

**Affiliations:** 1 Pediatric Cardiovascular Research Center, Cardiovascular Research Institute, Isfahan University of Medical Sciences, Isfahan, Iran; 2 Nursing and Midwifery Care Research Center, Department of Adult Health Nursing, Faculty of Nursing and Midwifery, Isfahan University of Medical Sciences, Isfahan, Iran; 3 Department of Epidemiology and Biostatistics, School of Health, Isfahan University of Medical Sciences, Isfahan, Iran

**Keywords:** Nursing, Internship, Students, Nursing process, Supervision Model, Clinical supervision

## Abstract

**Background:**

The nursing process is a systematic method for identifying the patient’s problems and planning to resolve them. It is also a crucial pillar of high-quality nursing care. Nursing internship students may lack the necessary skills to implement the nursing process due to the increased independence, the absence of constant professorial supervision, and limited experience. The clinical supervision model is a method of clinical education that bridges the gap between theory and practice.

**Objective:**

This study was conducted to investigate the impact of the clinical supervision model on the performance of nursing internship students in each of the five stages of the nursing process, as well as overall.

**Method:**

This experimental study was conducted in 2022. The 70 eligible internship students were conveniently selected and randomly assigned to either an intervention or a control group. In the present study, the clinical supervision model was implemented for the intervention group, while the control group received routine supervision. This was carried out over six sessions in three months. The data collection was conducted using a researcher-developed checklist of nursing process-based performance in both groups. Moreover, the Manchester questionnaire was used to evaluate the model in the intervention group. The variables considered as confounding factors included age, gender, marital status, number of monthly shifts, and grades of the nursing process credit completed in the third semester. SPSS version 16 software, descriptive statistics (frequency distribution, percentage, mean, and standard deviation), and analytical statistics (independent t-test, chi square, repeated measures Anova and LSD) were used to analyze the data.

**Results:**

Intergroup analysis revealed that there was no significant difference between the scores of nursing process steps and the total score before the intervention in the control and intervention groups, as well as in baseline characteristics (*P* > 0.05). According to the intragroup analysis, the intervention group showed a significant increase in both the total scores and scores of nursing process steps over time (*P* < 0.001), whereas the control group exhibited contradictory results (*P* > 0.05). Finally, the “P-Value Intervention” demonstrated the effectiveness of this training model in improving the performance of the intervention group based on the nursing process compared to the control group. The mean score of the Manchester questionnaire in the intervention group was 136.74, indicating the high impact of implementing the clinical supervision model in the intervention group.

**Conclusion:**

The results indicated that the implementation of the clinical supervision model led to improved utilization of the nursing process by nursing internship students at all stages. Therefore, it is recommended that nurse educators utilize the clinical supervision model by providing feedback on errors in action during supervision sessions to enhance the quality of nursing care provided by nursing internship students and improve patient safety in clinical environment.

**Supplementary Information:**

The online version contains supplementary material available at 10.1186/s12912-024-01840-0.

## Introduction

A nursing process is a dynamic approach to providing nursing care [1] and enhancing evidence-based nursing [2]. This process is recognized as a systematic method to identify the patient’s problems, plan to resolve them, implement care plans, and evaluate the extent to which the problems are solved [3]. The precise, scientific, and targeted implementation of the nursing process leads to complete and comprehensive patient care and facilitates the implementation of nursing interventions in the best way possible. The nursing process improves communication between nurses and healthcare members [4], results in satisfactory patient care [5],and saves time by reducing the care provision time [6].

Despite the advantages of the nursing process, its implementation in most hospitals, particularly in middle and low-income countries, is still a challenge [7]. According to the results of studies Ojewole (2017) et al. (2017) and Baraki et al. (2017), the majority of nurses employ an incomplete nursing process in their nursing care [8, 9]. Mamseri (2012) showed that although nurses receive training on the nursing process, it is infrequently used in the clinical setting [10]. Failure to implement the nursing process leads to blind obedience to physicians with no critical thinking, excessive dependence on them, low quality of care delivery, and reduced job satisfaction [11].

Also, the evidence shows that the nursing process is performed inefficiently by nursing students [3, 11, 12]. Atakro et al. (2019) conducted on nursing students to explore their experiences of clinical training show that failure to implement the nursing process in the clinical setting irritates them [13]. Nursing students’ failure to utilize the nursing process in providing scientific care and lack of decision-making power in planning and caring are among the significant problems of clinical education [14]. Among the main obstacles to non-implementation of the nursing process are the lack of familiarity with nursing process steps [15], lack of knowledge [16], lack of sufficient supervision [17],dependent and uncritical thinking [18], lack of clinical facilities, equipment, and a coherent and regular curriculum for training and evaluation of nursing students [19], limited educational opportunities [20], and lack of appropriate clinical training [9]. The studies of Oshvandi et al. (2013) and Zare et al. (2017) revealed that students do not acquire the necessary ability to apply the nursing process in actual conditions and perform their professional tasks [14, 21]. This issue is more significant for final-year nursing students (nursing internships) who, as a novice, experience the transition from being a student to a professional nurse since, during this period, faculty supervision reduces, and students are less experienced in facing clinical situations and experience high rates of stress [14, 22]. In this regard, Keshk et al., (2018) reported in their study that the scores of final-year nursing students’ (nursing internship) performance based on the nursing process before the educational intervention were very low [23].

According to Kestel et al. (2023), due to the weak performance of nursing students in implementing the nursing process, dynamic educational methods and active student participation are necessary to empower them and bridge the gap between theoretical and practical learning. This will effectively enable the use of the nursing process as a standard care method [24]. One educational model that combines theoretical and practical principles and plays an essential role in developing students’ abilities in clinical reasoning is the clinical supervision model. The concept of the clinical supervision model dates back to 1990 [25]. Clinical supervision can be defined as a process that focuse on providing empathic support to improve therapeutic skills, the transition of knowledge, and the facilitation of reflective practice [26]. In this model, individuals are guided and supported by a trained supervisor and receive feedback on their performance [27].

Clinical supervision provides the conditions for the student to meet the supervisor and deal with what is agreed on with the supervisor during routine supervision sessions and is an opportunity to share knowledge, experience, and clinical practice [28]. Knowledge sharing between the supervisor and student is one of the main features of this model [29]. Among the advantages of clinical supervision are increased satisfaction, self-efficacy, reduced stress and job burnout, increased quality of patient care, integration of theory and clinical issues, support for students seeking knowledge, reflective thinking and learning, and support for students’ emotional learning [27, 30, 31]. This model encourages students to provide safe patient care. In clinical supervision sessions, the supervisor uses clinical knowledge and experience to help nursing students improve their clinical performance. It is also imperative that the supervisor recognizes students’ progress during clinical supervision sessions [32]. The unique feature of this model is that, unlike other educational methods such as simulation, encouragement error, scenario-based problem which are typically conducted in a laboratory setting [33–35], it can be implemented in a real clinical environment. This model can be applied to the field of nursing process, which is closely integrated with the actual clinical environment [36]. The study by Snowdon et al. (2017) that investigated the impact of the clinical supervision model on improving the quality of nursing care showed that clinical supervision in a hospital setting increased the quality of care [37]. In Iran, Esfahani et al. (2016) conducted a study on the medication safety of ICU nurses using this method, and the results were positive [38]. According to another study that was conducted in Iran, clinical supervision model can be effective in improving the performance of nursing students in the nursing process [39].

The nursing internship project has been implemented in Iran for the first time in Isfahan since 2018. Based on this model, final-year nursing students undertake their internship course as a nursing internship for ten months in various hospital wards. The supervisor arranges 20 rotating shifts (morning, evening, and night) for the student in coordination with the supervisor and nursing internship professors. The nursing internship student is in charge of caring for 3–4 patients independently and under the supervision of the ward head nurse and nurses. The nurse educators assess nursing internship according to the schedule of the faculty during the nursing internship student’s attendance in the hospital. Considering the significance of implementing the nursing process at the bedside by students and nursing internship students’ inattention due to high stress and lack of experience, employing creative educational methods is indispensable. Given the role of the clinical supervision model in enhancing students’ self-confidence and bridging the gap between theory and practice, this study was conducted to investigate the effect of the clinical supervision model on nursing internship students’ nursing process-based performance in all five stages of the nursing process, as well as overall.

## Method

This experimental study with two control and intervention groups was conducted in the selected hospitals affiliated with Isfahan University of Medical Sciences in Isfahan, Iran in 2022. This study was single-blinded by a statistical analyst. The aim of the study was to examine the impact of the clinical supervision model on the performance of the nursing process by nursing internship students.

### Participants

Nursing internship students who were passing their last two semesters. The inclusion criteria included students’ willingness to participate in the study, obtaining a passing score in the nursing process credit during their third semester, and entering into the internship course. The exclusion criteria included the attendance of internship students in wards other than medical-surgical wards and a lack of willingness to continue participating in the study.

### Sample size

Considering that the aim of the current study was to compare the average scores between the control and intervention groups, the following formula was used, where S1 and S2 are the standard deviation of the scores of the intervention and control groups, respectively, and X1-X2 is the average difference between the two groups. Finally, the sample size in this study was considered to be 70 individuals with a power of 80%, a confidence level of 95%, a potential 10% attrition, and similar studies [23].

$$ {\text{N}} = {\left( {{{\text{Z}}_{1 - \frac{\alpha }{2}}} + {{\text{Z}}_{1 - \beta }}} \right)^2}\left( {{\text{S}}_1^2 + {\text{S}}_2^2} \right)/{\left( {{{\mathop {\text{X}}\limits^ - }_1} - {{\mathop {\text{X}}\limits^ - }_2}} \right)^2}$$

**Fig. 1:**
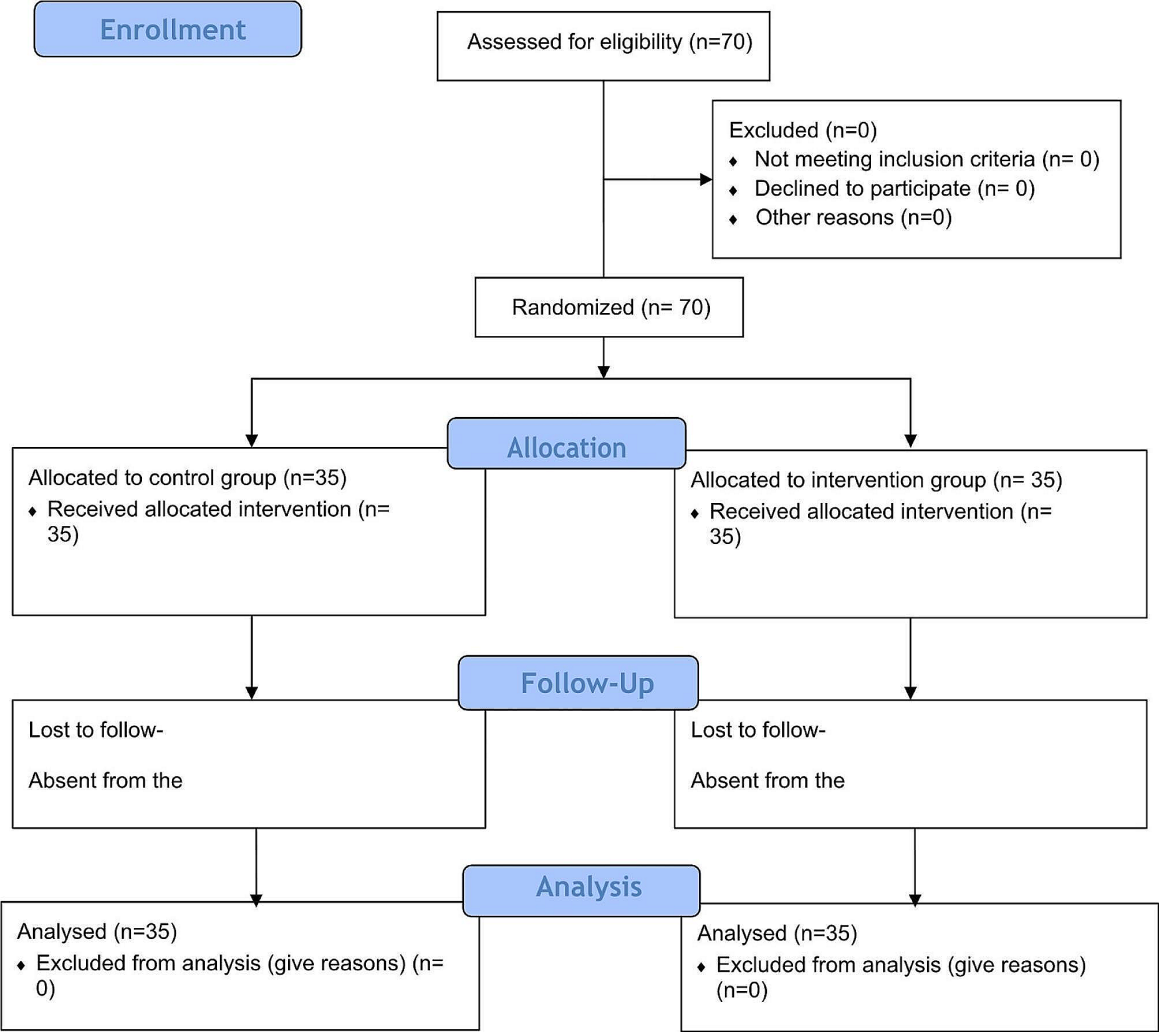
Consort flowchart

First, 70 volunteer internship students were selected through the convenience sampling method. Afterward, written and verbal consent was obtained from them. In the next step, seventy codes from 1 to 70 were assigned to them, and 35 individuals were assigned to each control and intervention group using random allocation method and random allocation software version 1.0.0. This software is based on a table of random numbers [40] (Fig. 1). This study was conducted in four hospitals in Isfahan city. The control group participants were placed in two hospitals, while the intervention group participants were placed in two other hospitals.

### Study tools

Data were collected using a demographic questionnaire, nursing process-based performance checklist and the Manchester Clinical Supervision Scale (MCSS). The demographic questionnaire included questions on the students’ age, gender, marital status, mean monthly shift, and nursing process mark in their third semester.

### Nursing process-based performance checklist

This checklist is researcher-made with 43 dichotomous items (yes and no) to check nursing internship students’ performance according to the nursing process. Gordon’s medical history form and accredited nursing resources, such as Carpentito, nursing care plan, and ALL-IN-ONE NURSING CARE PLANNING RESOURCE, were used to design this checklist, which consists of 5 sections: assessment, nursing diagnosis, planning, implementation, and evaluation. The assessment Sect. (27 items) includes quick assessment, physical examination, medications, lab tests, nutrition, visits, consultations, and scales related to falls, bedsores, and safety. The nursing diagnosis Sect. (4 items) includes a list of potential, actual, and possible nursing diagnoses according to the patient’s conditions and inscribing in the PES/PE format (Problem, Etiology, and Sign and Symptom). The planning Sect. (5 items) includes prioritizing nursing diagnoses and accurately inscribing measures, goals, expected outcomes, and planning for interventions. The implementation section includes 3 items: implementing measures with logical reasons and the participation of patients and assessing their responses. Finally, the evaluation section consists of 4 items, including collecting the results of the measures taken, comparing the patient’s answers with the expected outcomes, and applying changes in nursing diagnoses in case of failure to achieve the desired result(Supplementary file). The validation of checklist was done by asking the opinions of 15 experts with at least ten years of experience in the field of nursing process education, the scores of Content Validity Ratio, Content Validity Index, and Face Validity were obtained as 92, 80–100 and above 1.5, respectively. Regarding internal reliability, the Spearman-Brown coefficient was obtained as 0.75. Moreover, test-retest was used to examine the external reliability, and the ICC score of 0.675 and *P* < 0.001 were obtained.

### Manchester clinical supervision scale (MCSS)

This questionnaire, which assesses the effectiveness of the clinical supervision model, consists of 32 items with seven subscales, including trust and rapport, supervisor advice and support, improved care and skill, importance and value of CS, finding time, personal issues, and reflection scored on a 5-point Likert scale from 1 (totally disagree) to 5 (totally agree). This questionnaire was created in 1995 at the University of Manchester, England, and its validity and reliability were confirmed in Iran by Khani et al. (2009), and the effectiveness score was reported as 122 and more [41].

### Procedure

After obtaining the necessary permissions and the nursing internship students’ monthly shifts schedule from the Vice-Chancellor of Education of Isfahan Nursing School, the researcher referred to intended hospitals affiliated with Isfahan University of Medical Sciences, Isfahan, Iran. Before the intervention, students’ score for nursing process-based performance was checked in the control and intervention groups using the “nursing process-based performance” checklist by observing their performance. Clinical supervision sessions based on feedback and routine supervision sessions were held for the intervention and the control group, respectively.

### Intervention group

**Fig. 2:**
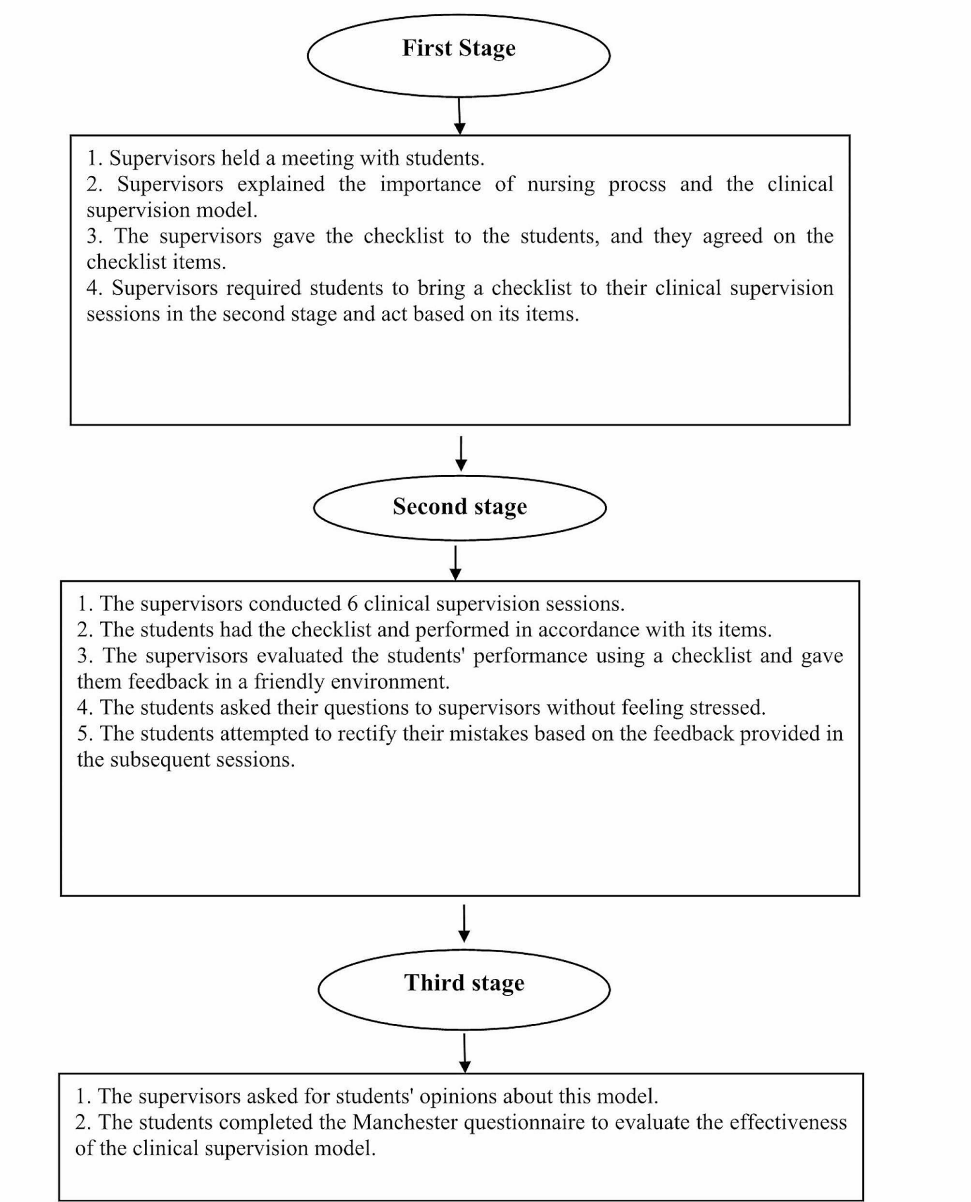
Description of the three stages of the clinical supervision model in the intervention group

The clinical supervision model consisting of three stages [38, 42] was implemented for the intervention group as follows (Fig. 2):

### First stage

In this study, two nurse educators with over 20 years of experience in supervising and teaching the nursing process were selected for supervision. They are competent in communication skills, giving feedback, and the nursing process. At this stage, supervisor held a one-on-one session outside the internship students’ shift schedule to alleviate their stress about missing out on their clinical practice. In this session, the importance and benefits of the nursing process and the harm followed by its defective implementation were discussed. Following that, the clinical supervision model, its benefits, steps, the supervisor’s and students’ duties, and the overall process were explained, the students’ questions were responded, ambiguities were resolved, and a conclusion was reached. Afterward, the checklist of performance based on the nursing process was provided to them, its items were discussed, and the students were requested to bring along the checklist during the supervision sessions in the second stage and perform according to its items. This stage lasted for a month.

### Second stage

At this stage, the supervisors attended the medical and surgical wards of the intended hospitals to hold supervision sessions. These sessions were held six times [43], fortnightly [44], during three months [27]. During these sessions, the students had the checklist and performed in accordance with its items. The supervisor would visit the patients’ bedside during the supervision sessions to assess the activities performed by the students in the five stages of the nursing process. Then, the supervisors provided them with feedback in case of any mistakes. Consequently, the students made an effort to rectify their previous errors during the subsequent supervision sessions and expressed their concerns to the supervisors. In each session, the supervisors recorded students’ scores on their performance based on the nursing process and the checklist. Each of these clinical supervision sessions lasted 40–60 min [28] and no other teaching methods were used in it.

### Third stage

At this stage, the supervisors attended the internal and surgical wards of the intended hospitals to evaluate the intervention. They and the students in the intervention group discussed clinical supervision, its strengths and weaknesses, and recommendations for improving the operationalization of this model. In addition, the Manchester questionnaire was used at this stage to determine the effectiveness of implementing the clinical supervision model. This stage lasted for a month.

### Control group

Similarly, in the control group, a one-on-one session outside of the students’ shift schedule was held by similar supervisors, and it was mentioned that six routine supervision sessions would be held fortnightly for three months, during which their nursing process-based performance would be checked. It should be noted that, unlike the intervention group, the students in the control group were not provided with a checklist [45]. During the supervision sessions, the supervisors checked their nursing process-based performance using the checklist and recorded the score of each session. However, they were not provided feedback on their erroneous performance except when the patient’s safety was threatened.

### Data analysis

The data was analyzed using SPSS version 16 software. Descriptive statistics, including frequency distribution, percentage, mean, and standard deviation, were employed. Analytical statistics, such as the independent t-test for comparing quantitative variables between two groups, chi-square for comparing qualitative variables between two groups, repeated measures ANOVA for comparing the scores related to the nursing process during the supervision sessions of the two control and intervention groups together and with each other, and LSD for comparing the mean scores of the nursing process in the intervention group two by two, were also utilized. A significance level of 0.05 was considered.

## Results

The number of study subjects in the control and intervention groups was 35 individuals. The normality of the data was confirmed using the results of the Kolmogorov-Smirnov test with *P* > 0.05. There was no significant difference between the control and intervention groups in terms of background characteristics (age, sex, marital status, number of monthly shifts, and grades of the nursing process credit completed in the 2nd semester), as well as the score of their nursing process-based performance before the intervention (Table 1).

**Table 1:** Differences in the baseline characteristics pre intervention between the intervention and control group

Variable	Mean ± SD^a^ or N (%)			P -Value ^b^	
		Intervention group	Control group		
Gender	Male	18 (51.4)	18 (51.4)	1.00	
	Female	17 (48.6)	17 (48.6)		
Marital status	Single	31 (88.6)	32 (91.4)	0.69	
	Married	4 (11.4)	3 (8.6)		
Mean shift		22.57 **±** 5.05	21.14 **±** 2.99	0.15	
Age		22.22 **±** 0.77	22.00 **±** 0.64	0.18	
Nursing process mark		16.22 **±** 1.60	15.96 **±** 1.53	0.48	
Nursing process total Score before Intervention		12.02 **±** 4.61	11.60 **±** 4.03	0.68	

The significance level is *p* ≤ 0.05

^a^ Standard Devision

^b^ Independent sample t- test and chi square

The Repeated Measures ANOVA showed that the scores of each step of the nursing process (assessment and recognition, nursing diagnosis, planning, implementation, and evaluation) and the total score were significant in the intervention group compared to the control group (P Value-Intervention < 0.001), and over time, the scores increased significantly in the intervention group (P Value-Time < 0.001). However, it was contradictory in the control group (P Value - Time > 0.05) (Table 2). Finally, a pairwise comparison of the scores for all five nursing process steps and the total score of the intervention group sessions, using the LSD test, revealed a significant increase in scores for each supervision session compared to the other sessions (*P* < 0.001).

**Table 2:** Components of Nursing Process scores of interventions and control group during clinical supervision sessions

Assesment Mark^a^			Nursing diagnoses Mark^b^			Planning Mark^c^		
Session	**Intervention group**	**Control group**	**Session**	**Intervention group**	**Control group**	**Session**	**Intervention group**	**Control group**
Before	9.42 ± 3.60	8.85 ± 3.08	**Before**	0.71 ± 0.66	0.77 ± 0.49	**Before**	0.71 ± 0.62	0.82 ± 0.38
First	11.74 ± 3.75	8.77 ± 3.06	**First**	1.08 ± 0.78	0.80 ± 0.72	**First**	1.08 ± 0.70	0.74 ± 0.44
Second	13.34 ± 3.46	9.11 ± 2.93	**Second**	1.57 ± 0.85	0.74 ± 0.44	**Second**	1.71 ± 0.78	0.71 ± 0.45
Third	15.98 ± 3.65	9.14 ± 2.80	**Third**	2.20 ± 0.86	0.71 ± 0.51	**Third**	2.31 ± 0.79	0.74 ± 0.44
Forth	17.08 ± 4.21	9.22 ± 2.79	**Forth**	2.68 ± 0.83	0.71 ± 0.51	**Forth**	3.05 ± 1.02	0.74 ± 0.44
Fifth	18.88 ± 4.10	9.25 ± 2.79	**Fifth**	3.11 ± 0.90	0.74 ± 0.5	**Fifth**	3.91 ± 0.91	0.77 ± 0.42
Six	20.22 ± 4.06	9.25 ± 2.68	**Six**	3.60 ± 0.65	0.77 ± 0.42	**Six**	4.65 ± 0.48	0.80 ± 0.47
P Value–Time^z^(effect size)	**< 0.001**(0.833)	0.266(0.038)	**P Value–Time** ^**z**^ **(effect size)**	**< 0.001**(0.802)	0.931(0.006)	**P Value–Time** ^**z**^ **(effect size)**	**< 0.001**(0.894)	0.721(0.015)
P Value–Interaction^z^ (effect size)	**< 0.001**(0.633)		**P Value–Interaction** ^**z**^ **(effect size)**	**< 0.001**(0.569)		**P Value–Interaction** ^**z**^ **(effect size)**	**< 0.001**(0.749)	
P Value– Intervention (effect size)^z^	**< 0.001**(0.488)		**P Value– Intervention** ^**z**^ **(effect size)**	**< 0.001**(0.674)		**P Value– Intervention** ^**z**^ **(effect size)**	**< 0.001**(0.760)	
Implemention Mark^d^			Evaluation Mark^e^			Total Mark^f^		
Session	Intervention group	Control group	**Session**	Intervention group	Control group	**Session**	Intervention group	Control group
Before	0.54 ± 0.050	0.57 ± 0.55	**Before**	0.42 ± 0.50	0.45 ± 0.56	**Before**	12.02 ± 4.61	11.60 ± 4.03
First	1.00 ± 0.59	0.65 ± 0.59	**First**	0.94 ± 0.59	0.54 ± 0.65	**First**	15.00 ± 5.59	11.65 ± 4.06
Second	1.31 ± 0.63	0.65 ± 0.59	**Second**	1.40 ± 0.73	0.57 ± 0.60	**Second**	18.62 ± 5.70	11.91 ± 3.99
Third	1.82 ± 0.89	0.60 ± 0.49	**Third**	1.97 ± 0.78	0.54 ± 0.56	**Third**	22.74 ± 5.95	11.42 ± 3.73
Forth	2.17 ± 0.56	0.62 ± 0.49	**Forth**	2.62 ± 0.84	0.57 ± 0.55	**Forth**	27.00 ± 6.27	11.60 ± 3.63
Fifth	2.54 ± 0.61	0.57 ± 0.50	**Fifth**	3.25 ± 0.74	0.60 ± 0.55	**Fifth**	31.00 ± 5.95	11.82 ± 3.74
Six	2.88 ± 0.40	0.60 ± 0.49	**Six**	3.71 ± 0.57	0.54 ± 0.50	**Six**	34.74 ± 5.98	11.94 ± 3.78
P Value–Time^z^(effect size)	**< 0.001**(0.759)	0.863(0.009)	**P Value–Time** ^**z**^ **(effect size)**	**< 0.001**(0.845)	0.793(0.013)	**P Value–Time** ^**z**^ **(effect size)**	**< 0.001**(0.917)	0.639(0.018)
P Value–Interaction^z^(effect size)	**< 0.001**(0.496)		**P Value–Interaction** ^**z**^ **(effect size)**	**< 0.001**(0.623)		**P Value–Interaction** ^**z**^ **(effect size)**	**< 0.001**(0.805)	
P Value–Intervention^z^(effect size)	**< 0.001**(0.677)		**P Value– Intervention** ^**z**^ **(effect size)**	**< 0.001**(0.729)		**P Value– Intervention** ^**z**^ **(effect size)**	**< 0.001**(0.609)	

The significance level is *p* ≤ 0.05, and statistically significant results are bolded / Maximum possible score a = 27, b = 4, c = 5, d = 3, e = 4, f = 43/ Repeated measure Anova test

The mean total score of the Manchester questionnaire in the present study was 136.74 from the perspective of the intervention group members, indicating the high impact of implementing this model (Table 3).

**Table 3:** Manchester clinical supervision scale– summed subscale and total scores

subscale	Possible score range	Actual score range	Mean summed score	S. D ^a^
Trust and rapport	6–30	23–29	25.88	1.62
Supervisor advice and support	5–25	17–24	20.80	1.89
Improved care and skill	7–35	26–33	29.85	1.88
Importance and value	4–20	13–20	17.20	1.58
Finding time	4–20	10–15	17.48	2.02
Personal issues	3–15	10–15	12.80	1.36
Reflection	3–15	10–15	12.71	1.40
Total score	32–160	129–150	136.74	5.23

^**a**^ Standard Devision

## Discussion

The nursing process is one of the significant components of the nursing profession, which has received less attention from nursing students, including nursing internships [23]. The nursing internship students’ knowledge is insufficient, and their performance score in implementing the nursing process has been reported as average [46]. Teixeira et al. (2016) in Portugal state in their study that clinical supervision sessions should be taken into account due to the gaps in the operationalization of the nursing process [47]. The present study aimed to investigate the impact of the clinical supervision model on the nursing process-based performance of nursing internship students in Isfahan, Iran. The results showed the positive effect of this model on nursing internship students’ nursing process-based performance.

The clinical supervisor influences the quality of clinical supervision sessions [48–50]. The present study employed clinical faculty members instead of nurses for clinical supervision. The results of the study by Lofmark et al. (2012), conducted in Norway to determine nursing students’ satisfaction with clinical professors and trained nurses’ supervision, showed that students were more satisfied with clinical professors because of the quality of education [51], since nurses lacked the necessary scientific knowledge to fully respond them [31]. They also lacked the opportunity to implement the nursing process at the bedside. Therefore, they were not expected to supervise students’ performance based on the nursing process [47].

In the first stage of the clinical supervision model in the intervention group, the items of the “Nursing process-based performance” checklist were agreed on with the students in the intervention group and provided to them. It was also mentioned that they were required to act based on the checklist items during the supervision sessions. The presence of a checklist during the supervision sessions helped students organize their work and provided guidance on how to approach patients and address their problems according to the stages of the nursing process. In the qualitative study by Thyness et al. (2022) involving medical students at two English and four Norwegian universities, students believed that the checklist was beneficial in preventing confusion and increasing discipline [52]. In this regard, based on the semi-experimental study conducted by Esfahani et al. (2016) in Iran, with only one intervention group and no control group, checklist-based practice improves intensive care nurses’ medication administration performance [38]. The study by Mirzaeipour et al. (2015) aimed to determine the impact of the action registration checklist on nurses’ performance in measuring central venous pressure in ICU wards, showed that the nursing care registration checklist could be an accessible, cost-effective, and straightforward method to improve nursing care [53]. The aforementioned studies all demonstrate the significance of utilizing a checklist and taking action on its items. In fact, the checklist provides structure for healthcare providers and helps prevent confusion and mistakes.

In the second stage of the clinical supervision model, six supervision sessions were conducted fortnightly for three months to coincide with the duration of nursing internship students’ attendance in medical-surgical wards, as indicated in studies [43, 44]. In the current study, continuous supervision had a positive and constructive effect on improving the performance of internship students in relation to the nursing process. Conducting continuous clinical supervision to establish a positive relationship between the clinical supervisor and the student is the most crucial factor in determining success in clinical practice [54]. At this stage, the supervisors attend to the wards and talk with the students about any problems they may have encountered. They prioritize solving these problems first. Afterward, the supervisor and student visit the patients’ bedsides together. All the actions that the students had taken based on the stages of the nursing process were reviewed based on the checklist items. According to students’ performance, supervisors were given feedback on students’ errors in a friendly envierment. The students calmly raised their questions and concerns with the supervisor and tried to correct their mistakes for the next supervision session. It was effective in increasing students’ learning motivation, quality of care, and critical thinking. One of the reported positive dimensions of the clinical supervision model is the joint dialogue between the supervisor and the supervisee and the feedback provided since they accelerate agreement and cooperation, challenge dominant ideologies, and change work practices, resulting in critical thinking [30]. The results of Plathe et al.‘s qualitative study (2021) on nursing students in their first year of a bachelor’s program in South-East Norway showed that feedback between students and professors in a friendly and stress-free environment led to improved student performance [55]. However, the type of feedback provided is important since inappropriate feedback can have contradictory results. The results of Clark et al.‘s study (2013), conducted in Nigeria, indicated that feedback could lead to tension and stress, as professors would threaten students during evaluations [56]. Therefore, maintaining a friendly environment is crucial for enhancing the quality of patient care during clinical supervision sessions.

Nursing internship students’ nursing process-based performance in the intervention group was significantly different compared to that of the control group in terms of the overall score and by steps in each clinical supervision session. In the study by Keshk et al. (2018) in Egypt, a training program, which was not detailed in the study, was implemented for internship students. Their individual and professional qualifications, nursing process skills, and individual abilities were measured using a self-made tool. The study found that students’ abilities increased in all steps of the nursing process [23]. According to the study by Dehghani et al. (2016) In Iran, clinical supervision resulted in a significant difference in the score of nursing students’ performance based on the nursing process after the intervention. This study was conducted on students up to the 7th semester. In addition to examining the nursing process, it investigated indicators of safety, infection control, and communication. The study reported positive effects from this model in all of these areas [39]. The results of ElZeneny’s study (2017), conducted in Cairo with nursing managers as supervisors, indicated that the quality of nursing care provided by nurses increased after implementing the clinical supervision model [57]. In the two studies mentioned above, as well as in the present study, a clinical supervision expert served as a supervisor, providing feedback to students and nurses based on a checklist in a friendly environment. These findings highlight the significance of having an experienced supervisor, utilizing detailed checklists, and providing feedback on incorrect performance in a friendly environment.

In the third stage, the Manchester questionnaire was used to investigate the effectiveness of clinical supervision in the intervention group. The results indicated the high effectiveness of this model in this group. In Dawson et al.‘s (2012) study conducted in Australia, the level of satisfaction with and effectiveness of the clinical supervision model was found to be adequately high, as indicated by the Manchester questionnaire [44]. Similarly, a study in England investigated clinical supervision sessions lasting more than one hour monthly. The study found that 75% of nurses viewed the sessions positively and considered clinical supervision a valuable source for developing skills and competencies [58]. The aforementioned studies demonstrate the efficacy of the clinical supervision model as perceived by nurses and nursing students. This is attributed to the provision of constructive feedback in a friendly environment by their supervisors.

## Conclusions

The nursing process is one of the factors affecting the quality of nursing care and improving patient safety. In order to reduce the gap between theory and practice, novel teaching methods should be employed for nursing, particularly internship students, who enjoy more independence and are less supervised clinically. In this study, the use of a clinical supervision model helped bridge the gap between theory and practice. The involvement of expert supervisors who provided feedback to students in a friendly environment resulted in improved performance in all stages of the nursing process. The most significant improvement was observed in the nursing diagnosis and implementation stages. Indeed, the clinical supervision model allows for the application of classroom learning in a real clinical environment. The evaluation of the clinical supervision model in the third stage also showed that the students found the implementation of the model to be effective in applying the nursing process. It is evident that the implementation of the nursing process results in more evidence-based actions and care, reducing errors and ultimately enhancing patient safety. Therefore, it is possible to teach faculty how to supervise students based on the clinical supervision model, provide feedback, and evaluate them according to their performance using valid checklists that are accepted by students in the clinical environment. Finally, professors who learn and apply the clinical supervision model in their clinical supervision sessions can improve the quality of nursing care provided by nursing internship students.

## Limitations

One of the limitations of this study was that other professors supervised the internship students to grade the course credit and their visit method was not based on the clinical supervision model. Another limitation was these students’ failure to attend the medical-surgical ward for three consecutive months due to their attendance in other wards (such as midwifery or pediatrics) but the basis of the clinical supervision model is continuous supervision. The last limitation of this study was the inclusion of students from different hospitals, resulting in varying environmental conditions among the samples. But all of these limitations were largely moderated through random allocation and the presence of a control group.

### Implication

The nursing process is one of the most important pillars of the nursing profession and gives it its identity. Ignoring the nursing process is a challenging issue, especially in developing countries. So, new educational methods should be used to implement it. The clinical supervision model is a method that can be implemented and used in real clinical environments. Therefore, it is preferable to other educational methods that can only be implemented in laboratory environments. In this method of learning, nurses and nursing students in the clinical environment realize their mistakes and strive to correct them. In fact, this educational method helps nurses to study more and implement what they have learned in the classroom in a clinical setting. It also facilitates independent learning. All of this leads to improved and more evidence-based care.

It is suggested to conduct a qualitative study in various areas of clinical supervision, such as feedback, duration of meetings, and checklists. Particularly, using the focus group method to explore the strengths and weaknesses of the clinical supervision model. It is also recommended to apply this model to nurses with continuous supervision meetings and without routine visits to accurately assess the clinical supervision model.

### Electronic supplementary material

Below is the link to the electronic supplementary material.

Supplementary Material 1

## Data Availability

The data supporting the findings of this study are available on request from the corresponding author. The data are not publicly available due to privacy or ethical restrictions.

## References

[1] Mbithi BW, Mwenda CS, Karonjo J (2018). Observed utilization of the nursing process among nurses in selected public health care facilities in Kenya. Int J Nurs.

[2] Movlavi S, Salehi S (2021). Examining the effect of implementation of the nursing process on students’ health behaviors. Int J Adolesc Med Health.

[3] Nes AA, Høybakk J, Zlamal J, Solberg MT (2021). Mixed teaching methods focused on flipped classroom and digital unfolding case to enhance undergraduate nursing students’ knowledge in nursing process. Int J Educational Res.

[4] Ghanbari A, Monfared A, Hoseinzadeh T, Moaddab F, Sedighi A (2017). The impact of the nursing process education on critical thinking of nursing students. Res Med Educ.

[5] Yildirim B, Ozkahraman S (2011). Critical thinking in nursing process and education. Int J Humanit Social Sci.

[6] Habibzadeh H, Khajehali N, Khalkhali H, Mohammadpour Y (2013). Effect of evidence-based nursing training on nursing students ability in executive skill of nursing process in Urmia University of Medical Sciences, 2013. Nurs Midwifery J.

[7] Mahmoud MH, Bayoumy HM (2014). Barriers and facilitators for execution of nursing process from nurses’ perspective. Int J Adv Res.

[8] Ojewole FO, Samole AO (2017). Evaluation of the nursing process utilization in a teaching hospital, Ogun State, Nigeria. J Nurs Midwifery Sci.

[9] Baraki Z, Girmay F, Kidanu K, Gerensea H, Gezehgne D, Teklay H (2017). A cross sectional study on nursing process implementation and associated factors among nurses working in selected hospitals of Central and Northwest zones, Tigray Region, Ethiopia. BMC Nurs.

[10] Mamseri RA. The nursing process as a means of improving patient care. 2012.

[11] Sayadi N, Rokhafroz D (2013). Nursing students’ perspectives about a mobile software on nursing process for bedside use. Iran J Med Educ.

[12] Munangatire T, Nambuli SM (2022). Nursing students’ perceptions and experiences of utilising the nursing process at a university teaching hospital in Namibia. Int J Afr Nurs Sci.

[13] Atakro CA, Armah E, Menlah A, Garti I, Addo SB, Adatara P, Boni GS (2019). Clinical placement experiences by undergraduate nursing students in selected teaching hospitals in Ghana. BMC Nurs.

[14] Oshvandi K, Bikmoradi A (2013). The effects of Inquiry-based clinical instruction of nursing students on applying nursing process skill. Avicenna J Nurs Midwifery Care.

[15] de Moraes Lopes MHB, Higa R, Dos Reis MJ, De Oliveira NR, Christóforo FFM (2010). Evaluation of the nursing process used at a Brazilian teaching hospital. Int J Nurs Terminologies Classifications.

[16] Ofi B, Sowunmi O (2012). Nursing documentation: experience of the use of the nursing process model in selected hospitals in I badan, o yo S tate, N igeria. Int J Nurs Pract.

[17] Gazari T, Apiribu F, Afaya RA, Awenabisa AG, Dzomeku VM, Mensah ABB, Amooba PA, Kukeba MW (2021). Qualitative exploration of the challenges and the benefits of the nursing process in clinical practice: a study among registered nurses in a municipal hospital in Ghana. Nurs Open.

[18] Yousefy A, reza Yazdannik A, Mohammadi S (2015). Exploring the environment of clinical baccalaureate nursing students’ education in Iran; a qualitative descriptive study. Nurse Educ Today.

[19] Amini A, Bayat R, Amini K (2020). Barriers to clinical education from the perspective of nursing students in Iran: an integrative review. Archiv Pharm Pract.

[20] Mamaghani EA, Rahmani A, Hassankhani H, Zamanzadeh V, Campbell S, Fast O, Irajpour A (2018). Experiences of Iranian nursing students regarding their clinical learning environment. Asian Nurs Res.

[21] Zare A, Kargar JM. The Effect of the nursing process education according to the concept map on learning of the nursing process. 2017;6(4):65–70. 10.21859/jne-06048.

[22] Abdelsalam G, Basal A, Ebrahem R, Elnagar S (2016). Perceptions of role transition among nursing interns at Tanta University. J Nurs Health Sci.

[23] Keshk LI, Qalawa SAA, Ibrahim N (2018). Effectiveness of an educational program regarding nursing process on acquiring advanced skills among internship nursing students. Int J Nurs.

[24] Kestel S, Korkmaz F (2023). Effectiveness of blended learning in nursing process teaching: first-year nursing students. Teach Learn Nurs.

[25] Crafoord M, Fagerdahl A (2017). Clinical supervision in perioperative nursing education in Sweden-A questionnaire study. Nurse Educ Pract.

[26] Winstanley J, White E (2003). Clinical supervision: models, measures and best practice. Nurse Researcher (through 2013).

[27] Gonge H, Buus N (2015). Is it possible to strengthen psychiatric nursing staff’s clinical supervision? RCT of a meta-supervision intervention. J Adv Nurs.

[28] Butterworth T (2022). What is clinical supervision and how can it be delivered in practice. Nurs Times [online].

[29] Honkavuo L (2020). Nursing students’ perspective on a caring relationship in clinical supervision. Nurs Ethics.

[30] Dilworth S, Higgins I, Parker V, Kelly B, Turner J (2013). Finding a way forward: a literature review on the current debates around clinical supervision. Contemp Nurse.

[31] Hall-Lord ML, Theander K, Athlin E (2013). A clinical supervision model in bachelor nursing education–purpose, content and evaluation. Nurse Educ Pract.

[32] Gopee N. Mentoring and supervision in healthcare. Sage; 2015.

[33] Kim K, Lee I (2020). Medication error encouragement training: a quasi-experimental study. Nurse Educ Today.

[34] Jarvill M, Jenkins S, Akman O, Astroth KS, Pohl C, Jacobs PJ (2018). Effect of simulation on nursing students’ medication administration competence. Clin Simul Nurs.

[35] Kuo S-Y, Wu J-C, Chen H-W, Chen C-J, Hu SH (2020). Comparison of the effects of simulation training and problem-based scenarios on the improvement of graduating nursing students to speak up about medication errors: a quasi-experimental study. Nurse Educ Today.

[36] Heaven C, Clegg J, Maguire P (2006). Transfer of communication skills training from workshop to workplace: the impact of clinical supervision. Patient Educ Couns.

[37] Snowdon DA, Leggat SG, Taylor NF (2017). Does clinical supervision of healthcare professionals improve effectiveness of care and patient experience? A systematic review. BMC Health Serv Res.

[38] Esfahani AK, Varzaneh FR, Changiz T (2016). The effect of clinical supervision model on high alert medication safety in intensive care units nurses. Iran J Nurs Midwifery Res.

[39] Dehghani M, Ghanavati S, SOLTAN B, Aghakhani N, Haghpanah S (2016). Impact of clinical supervision on field training of nursing students at Urmia University of Medical Sciences. J Adv Med Educ Professionalism.

[40] Saghaei M (2004). Random allocation software for parallel group randomized trials. BMC Med Res Methodol.

[41] Khani A, Jaafarpour M, Jamshidbeigi Y (2009). Translating and validating the Iranian version of the Manchester Clinical Supervision Scale (MCSS). J Clin Diagn Res.

[42] Nasiriani K, Salami T, Dehghani H. Clinical supervision in nursing education: definitions and models. 2013; 13(3):179–187.

[43] Edwards D, Cooper L, Burnard P, Hanningan B, Adams J, Fothergill A, Coyle D (2005). Factors influencing the effectiveness of clinical supervision. J Psychiatr Ment Health Nurs.

[44] Dawson M, Phillips B, Leggat SG (2012). Effective clinical supervision for regional allied health professionals–the supervisee’s perspective. Aust Health Rev.

[45] Shahzeydi A, Farzi S, Tarrahi MJ, Babaei S. The effect of clinical supervision model on nursing interns medication safety competence and knowledge: a clinical trial.Nursing and midwifery studies, 2023, 12(4): 190–6. 10.48307/NMS.2023.412822.1251.

[46] Iskin M, Abay B (2022). The achievements gained by nursing students from Internship practices: a descriptive study. Int J Caring Sci.

[47] Teixeira SMM, Carvalho AL, Cruz S (2016). Self-care assessment as an indicator for clinical supervision in nursing. Revista Da Rede De Enfermagem do Nordeste.

[48] Wong LC, Wong PT, Ishiyama FI (2013). What helps and what hinders in cross-cultural clinical supervision: a critical incident study. Couns Psychol.

[49] Mayton H. Being your authentic self: an exploration of the relationship between authenticity and self-efficacy in counselor trainees. The University of North Carolina at Greensboro; 2018.

[50] Maplethorpe F, Dixon J, Rush B (2014). Participation in clinical supervision (PACS): an evaluation of student nurse clinical supervision facilitated by mental health service users. Nurse Educ Pract.

[51] Löfmark A, Thorkildsen K, Råholm M-B, Natvig GK (2012). Nursing students’ satisfaction with supervision from preceptors and teachers during clinical practice. Nurse Educ Pract.

[52] Thyness C, Steinsbekk A, Grimstad H (2022). Learning from clinical supervision–a qualitative study of undergraduate medical students’ experiences. Med Educ Online.

[53] Mirzaeipour F, Imanipour M, Shahsavari H, Haghani H, Hazaryan M (2015). Effect of checklist application on performance of intensive care nurses in measuring central venous pressure. Hayat J.

[54] Bourke-Matas E, Maloney S, Jepson M, Bowles K-A (2020). Evidence-based practice conversations with clinical supervisors during paramedic placements: an exploratory study of students??? Perceptions. J Contemp Mediacal Educ.

[55] Plathe H, Solheim E, Eide H (2021). Nursing students’ and Preceptors’ experiences with using an Assessment Tool for Feedback and Reflection in Supervision of Clinical skills: a qualitative pilot study. Nurs Res Pract.

[56] Clark A, Olumese H (2013). Effective supervision as a challenge in technical and vocational education delivery: ensuring quality teaching/learning environment and feedback mechanism. Basic Res J Educ Res Rev.

[57] Elzeneny SR, Seada AM, El AleamEtewy E (2017). Effect of clinical supervision training program for nurse managers on quality of nursing care in intensive care units. Int J Nurs Didactics.

[58] Carney S (2005). Clinical supervision in a challenging behaviour unit. Nurs Times.

