# Supplementary material for: The effect of the clinical supervision model on nursing internship students’ nursing process-based performance: an experimental study
Source: BMC Nurs. 2024 Mar 8;23:166. doi: 10.1186/s12912-024-01840-0 (PMC10921759; doi:10.1186/s12912-024-01840-0)
Supplement: Supplementary file 1 — Supplementary Material 1 [file 12912_2024_1840_MOESM1_ESM.docx]

**"Nursing Process-Based Performance Checklist" For Nursing Internship Students**

| **Steps of the Nursing Process** | **Items** | **Yes** | **No** |
| --- | --- | --- | --- |
| **Assessment** | 1. The student introduced himself/herself to the patient. |  |  |
|  | 2. The student is aware of the patient's chief complaint. |  |  |
|  | 3. The student has enough information about the patient's present illness. |  |  |
|  | 4. The student is aware of the underlying diseases of the patient. |  |  |
|  | 5. The student has examined the patient's allergies. |  |  |
|  | 6. The student has examined the patient's smoking habits. |  |  |
|  | 7. The student has examined the patient's daily activity level. |  |  |
|  | 8. The student has examined the patient's airway. |  |  |
|  | 9. The student has examined the patient's breathing. |  |  |
|  | 10. The student has examined the patient's circulation. |  |  |
|  | 11. The student has examined the patient's catheters, including Foley catheter, NG tube, chest tube, IV line, and others. |  |  |
|  | 12. The student has calculated the Braden score of the patient. |  |  |
|  | 13. The student has calculated the Morse score of the patient. |  |  |
|  | 14. The student has calculated the patient's Geneva score. |  |  |
|  | 15. The student has calculated the patient's Wells score. |  |  |
|  | 16. The student has examined the patient's pain pattern. |  |  |
|  | 17. The student monitored the patient's vital signs during him/her shift. |  |  |
|  | 18. The student is aware of the daily medical orders prescribed for the patient. |  |  |
|  | 19. The student has examined patient requested consultations. |  |  |
|  | 20. The student has examined the patient's elimination-excretion pattern. |  |  |
|  | 21. The student has examined the patient's sleep pattern. |  |  |
|  | 22. The student has examined the patient's nutritional pattern. |  |  |
|  | 23. The student has examined the patient's fluid intake and output. |  |  |
|  | 24. The student is aware of the results of the patient's lab tests. |  |  |
|  | 25. The student is aware of his/her patient's medications. |  |  |
|  | 26. The student has examined medication and food interactions. |  |  |
|  | 27. According to the patient's disease, the student knows which physical examination to perform. |  |  |
| **Nursing diagnosis** | 28. The student wrote nursing diagnoses correctly (based on PES/PE). |  |  |
|  | 29. The student has listed potential nursing diagnosis. |  |  |
|  | 30. The student has listed actual nursing diagnosis. |  |  |
|  | 31. The student has listed possible nursing diagnosis. |  |  |
| **Planning** | 32. The student has prioritized nursing diagnosis. |  |  |
|  | 33. The student correctly expresses the goal of nursing diagnosis based on the problem. |  |  |
|  | 34. The student has listed the nursing diagnosis interventions according to the reference, based on etiology. |  |  |
|  | 35. The student correctly identified the expected outcomes of nursing diagnosis based on signs and symptoms. |  |  |
|  | 36. The student has developed a correct and accessible plan based on nursing diagnosis. |  |  |
| **Implementation** | 37. The student has implemented the interventions based on the nursing diagnosis and with scientific rationale. |  |  |
|  | 38. The student has implemented the interventions with the participation of the patient, family, and healthcare providers. |  |  |
|  | 39. The student has examined the patient's response to the interventions implemented. |  |  |
| **Evaluation** | 40. The student has collected the results of all the interventions that were implemented. |  |  |
|  | 41. The student has compared the patient's response to the implementation taken with the expected outcomes. |  |  |
|  | 42. The student has completed the evaluation process with the participation of the patient, family, and healthcare providers. |  |  |
|  | 43. After the evaluation, if the desired result is not achieved, the student has made changes in goal, expected outcome, and interventions. |  |  |
